# Gap-Junction Communication Pathways in Germinal Center Reactions

**DOI:** 10.1155/1998/45913

**Published:** 1998

**Authors:** Tibor Krenacs, Martin Rosendaal

**Affiliations:** 1 Department of Anatomy and Developmental Biology University College London UK; 2 Department of Pathology Albert Szent-Gyorgyi Medical University Szeged Hungary

**Keywords:** Connexin43, gap junctions, germinal center, immune response, intercellular communication

## Abstract

Intercellular channels called gap junctions enable multicellular organisms to exchange information rapidly between cells. Though gap junctions are held to be ubiquitous in solid tissues, we have only recently found them in the lymphoid organs. Functional direct cell-cell communication has now been confirmed by us and other groups in bone marrow, thymus, and in secondary lymphoid tissues. What functions do they serve in the lymphoreticular system where, so far, only cytokines/growth factors and adhesion molecules have been considered as regulators? Here we show evidence for and refer to published work about functional direct cellcell communication through gap junctions in germinal center reactions and make proposals for their role in the immune response.

We found a large amount of the connexin43 (Cx43) gap junctions in the germinal centers of secondary lymphoid follicles. Ultrastructurally and immunohistologically, most of the junctions were detected on the processes of follicular dendritic cells (FDC) enveloping nondividing centrocytes in the light zone of germinal centers where B-cell selection is thought to take place. Further support for this finding came by revealing the Cx43 mRNA *in situ* at the same location as the protein. On antigen challenge, gap junctions appeared on the FDC as they formed meshworks in germinal centers. In order to find out which germinal center cells communicate directly, we separated FDC-rich, low-density, B-cell fractions from human tonsil. In culture, we injected single FDC with the low-molecular-weight fluorescent dye, Lucifer Yellow (Mr 457 Da), which passed between adjacent FDC and sometimes from FDC to B cells.

Based on these findings and their assigned functions in other tissues, gap junctions may contribute to germinal center reactions in the following ways: (1) they may regulate follicle pattern formation by controlling FDC growth, (2) they may be involved in FDC-B-cell signaling contributing to the final rescue of selected B cells from apoptosis, and (3) they may enable FDC to work as a functional syncytium providing a cellular internet for integrating germinal center events. Data supporting these interpretations are briefly discussed.


*Developmental Immunology*, 1998, Vol. 6, pp. 111–118
Reprints available directly from the publisher
Photocopying permitted by license only

© 1998 OPA (Overseas Publishers Association)
N.V. Published by license under the
Harwood Academic Publishers imprint,
part of the Gordon and Breach Publishing Group
Printed in Malaysia

### Gap-Junction Communication Pathways in Germinal Center Reactions

TIBOR KRENACS[†] and MARTIN ROSENDAAL[*]

[a]*Department of Anatomy and Developmental Biology, University College London, England*

( *In final form 30 May 1997*)

Intercellular channels called gap junctions enable multicellular organisms to exchange information rapidly between cells. Though gap junctions are held to be ubiquitous in solid tissues, we have only recently found them in the lymphoid organs. Functional direct cell-cell communication has now been confirmed by us and other groups in bone marrow, thymus, and in secondary lymphoid tissues. What functions do they serve in the lymphoreticular system where, so far, only cytokines/growth factors and adhesion molecules have been considered as regulators? Here we show evidence for and refer to published work about functional direct cell-cell communication through gap junctions in germinal center reactions and make proposals for their role in the immune response.

We found a large amount of the connexin43 (Cx43) gap junctions in the germinal centers of secondary lymphoid follicles. Ultrastructurally and immunohistologically, most of the junctions were detected on the processes of follicular dendritic cells (FDC) enveloping nondividing centrocytes in the light zone of germinal centers where B-cell selection is thought to take place. Further support for this finding came by revealing the Cx43 mRNA *in situ* at the same location as the protein. On antigen challenge, gap junctions appeared on the FDC as they formed meshworks in germinal centers. In order to find out which germinal center cells communicate directly, we separated FDC-rich, low-density, B-cell fractions from human tonsil. In culture, we injected single FDC with the low-molecular-weight fluorescent dye, Lucifer Yellow ($M_r$ 457 Da), which passed between adjacent FDC and sometimes from FDC to B cells.

Based on these findings and their assigned functions in other tissues, gap junctions may contribute to germinal center reactions in the following ways: (1) they may regulate follicle pattern formation by controlling FDC growth, (2) they may be involved in FDC-B-cell signaling contributing to the final rescue of selected B cells from apoptosis, and (3) they may enable FDC to work as a functional syncytium providing a cellular internet for integrating germinal center events. Data supporting these interpretations are briefly discussed.

*Keywords:* Connexin43, gap junctions, germinal center, immune response, intercellular communication

### INTRODUCTION

T-cell-dependent antigen response is held to be regulated by receptor-ligand interactions such as those among antigen, antibody, cytokines, or adhesion molecules and their cell-membrane receptors (see Clark and Ledbetter, 1994; MacLennan, 1994; Thorbecke et al., 1994). Recently, we found evidence that a direct regulatory pathway via gap junctions also exists in lymphoid germinal centers where plasma and

*Corresponding author.
†Present address: Department of Pathology, Albert Szent-Gyorgyi Medical University, Szeged, Hungary memory B-cell production takes place (Krenacs and Rosendaal, 1995; Krenacs et al., 1997).

Gap junctions consist of hundreds of small channels (connexons) arranged into plaques in the cell membrane (Makowski et al., 1977). Connexons produced by adjacent cells are aligned and small molecules and ions (< 1 kDa) can be rapidly exchanged through them between the coupled cells. These may include small metabolites, morphogens, and second messengers like $Ca^{+2}$ ions, cyclic-AMP, or 1,4,5-inositol-triphosphate (Saez et al., 1989; Granot and Dekel, 1994). Metabolic communication through gap junctions has been implicated in the regulation of cell growth and proliferation during tissue development (Loewenstein and Rose, 1992; Warner, 1992) and in the rapid propagation of local signals through coupled cell networks (reviewed by Bruzzone et al., 1996). Members of the gap-junction multigene family can be traced in tissues immunohistologically by detecting connexin molecules, six of which form the connexon hemi-channel (Makowski et al., 1977; Kumar and Gilula, 1996).

In germinal center reactions, combinations of the antigen, T-cell cytokines, growth factors, and adhesion molecules are implicated in the activation, rescue, selection, and isotype switching of B lymphocytes. As a result, plasma and memory B cells are formed in a highly controlled manner against the antigen (Clark and Ledbetter, 1994; MacLennan, 1994; Thorbecke et al., 1994). Considering their individual complexity, it is not clear how these multifactorial events are so integrated that the whole immune response seems strictly organized both structurally and functionally. It has long been observed that the microenvironment of FDC plays a crucial role in T-cell-dependent antigen response. The FDC meshwork, formed on antigen challenge attracts all activated B cells and assists them in meeting factors they need for their final maturation. Though FDC are probably not autonomous they seem to play a significant role in integrating germinal center events. In this paper we speculate on how FDC function is coordinated and which kinds of interaction take place between FDC and lymphocytes, in the light of our recent finding of direct intercellular communication through gap junctions in the lymphoid germinal center.

### RESULTS AND DISCUSSION

### Gap-Junction Expression in Lymphoid Organs

Of the three most prevalent gap-junction types, Cx43, -32, and -26, only Cx43 was found in the lymphoreticular system, that is, in bone marrow (Rosendaal et al., 1991, 1994), thymus (Alves et al., 1995), and secondary lymphoid organs (Krenacs and Rosendaal, 1995). The remarkable feature of gap-junction expression in spleen, lymph nodes, and MALT (the mucosa associated lymphoid tissue) is the presence of large numbers of Cx43 gap junctions in germinal centers of fully formed secondary follicles (Figure 1A). Transmission and freeze-fracture electron microscopy and immunohistochemistry revealed that most junctions are produced by FDC, which also express CD21, CD35 complement receptors, and form desmosomal junctions (Krenacs and Rosendaal, 1995; Krenacs et al., 1997) (Figure 1B). The number of FDC and gap junctions shows a strong positive correlation, being densest in the light zone (Figure 2), where B-cell selection is held to happen (Lindhout et al., 1993). Here, gap junctions are arranged along the processes of FDC that envelope nondividing centrocytes. Mice immunization experiments confirm that developing FDC produce gradually increasing numbers of gap junctions up to the formation of full secondary follicles (Krenacs et al., 1997). Two of the interesting questions these findings raise are: (1) Why do FDC need to produce lots of gap junctions when they form germinal centers? (2) Do only FDC utilize gap-junctional communication or is this pathway also part of FDC-B-cell interactions?

### Cell Coupling in the Germinal Center

To find out whether the presence of gap junctions means functional coupling between germinal center cells, we set up FDC-B-cell cultures from human tonsils. Separated FDC soon started forming gap junctions in their membranes as they developed

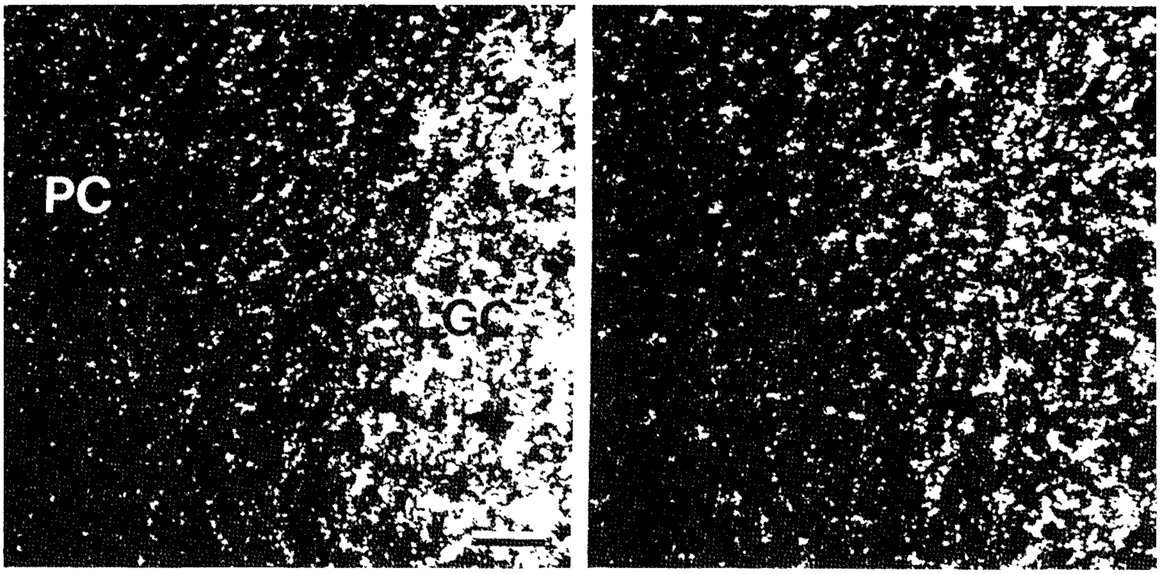

FIGURE 1 Immunofluorescence co-localization of (A) Cx43 gap junctions and (B) desmosomal junctions (desmoplakin reaction) in a human tonsil section. Note the same distribution of both types of junction with strong expression in the germinal center (GC; right half of the images) and gradual downregulation toward the paracortex (PC; left half of the images). Bar: 20 $\mu$m.

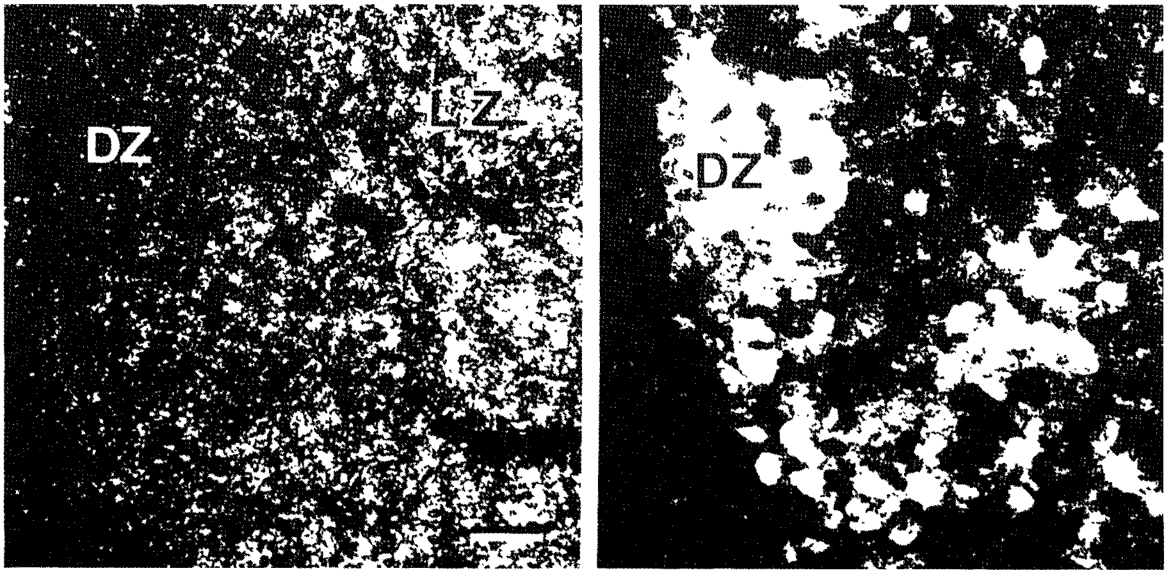

FIGURE 2 Simultaneous immunofluorescence for (A) Cx43 and (B) Ki-67 antigen in a tonsillar germinal center. Cx43 gap junctions are concentrated in the light zone (A; LZ), where B centrocytes are hardly labeled for the proliferation marker antigen Ki-67 (B). There are only few junctions in the dark zone (DZ) full of proliferating centroblasts. Bar: 20 $\mu$m.

projections toward B cells from the beginning of culture (Figure 3). Gap junctions were sometimes in close association with B cells and the enveloping FDC processes. This was most obvious in FDC-B-cell clusters in later cultures (Figure 4). The intimate relationship between FDC, B cells, and gap junctions was very similar to the tissue findings (see Figure 1A) and raised the possibility of heterologous communication between these cells. We used dye-transfer studies and established that this was the case (Krenacs et al., 1997). We microinjected elongated FDC with a small-molecular-weight fluorescent dye Lucifer Yellow, which passes through gap junctions. In a few minutes, one to three neighboring FDC lit up and sometimes the dye was also delivered to B cells through gap junctions. Besides FDC, some isolated germinal center B cells expressed the gap-junction protein too (Figure 5).

### Function for Gap Junctions

For assessing the potential of direct cell-cell communication in the germinal center, it is useful to outline briefly what functions have been attributed to gap junctions elsewhere. That gap junctions serve important function(s) is expected from their ubiquitous presence in multicellular organisms (Kumar and Gilula, 1996) and because their channel-forming connexins are highly conserved throughout evolution (Warner, 1992). Functional data collected so far can be grouped along two main lines. On one hand, gap junctions provide a low-resistance pathway for the rapid propagation of local signals through coupled cell networks (Bruzzone et al, 1996). Depolarizing waves are held to spread in this way between excitable cells such as cardiac myocytes (Rook et al., 1990), smooth muscle cells (Miller et al., 1989), or neurones (Bruzzone et al., 1996), resulting in rapid and coordinated responses. In nonexcitable tissues, local stimuli may generate oscillating $Ca^{+2}$ waves across coupled cells. In this way, gap-junctional communication is involved, for instance, in the integration of insulin secretion in the endocrine pancreas (Vozzi et al., 1995) or in the propagation of vasopressin action between hepatocytes (Nathanson et al., 1995).

On the other hand, gap junctions are thought to provide a pathway for growth regulatory signals and

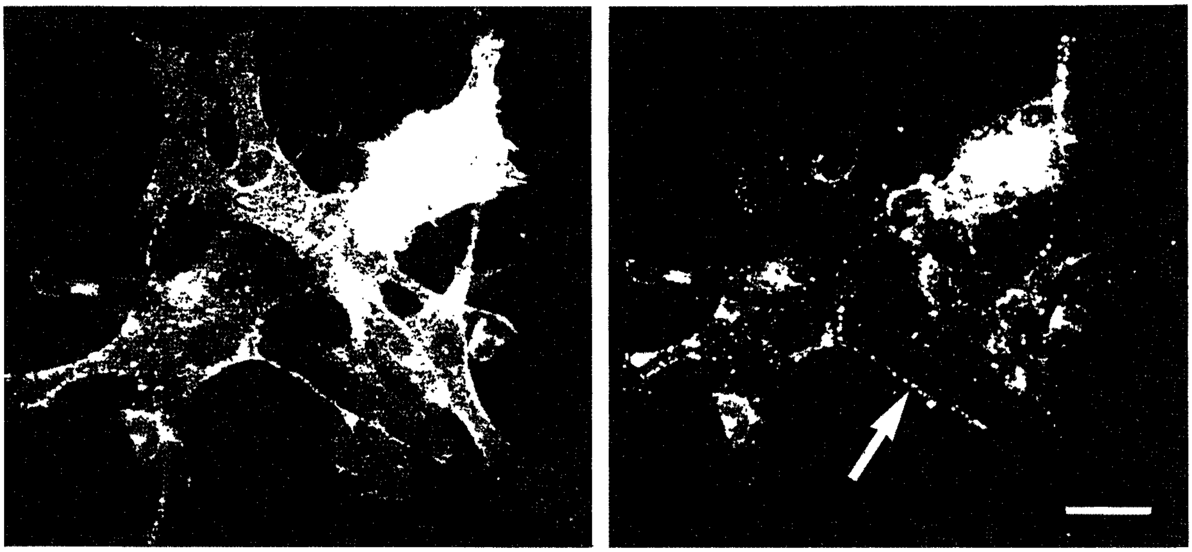

FIGURE 3 FDC-B-cell culture simultaneously immunostained for (A) CD35 and (B) Cx43 after 4 hr of culture. Sheets and narrow processes of FDC decorated with immunolabeled gap junctions (B; arrow) are extending toward and enveloping B lymphocytes (A). Bar: 10 $\mu$m.

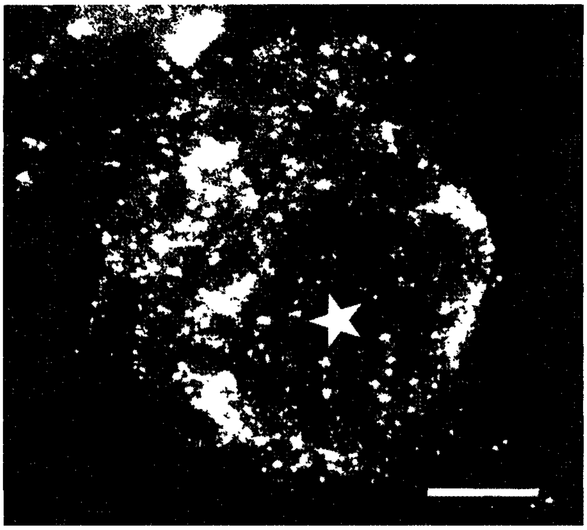

FIGURE 4 FDC-B-cell cluster consisting of 2 to 4 FDC and 15 to 20 B cells in a 6-hr culture is heavily labeled for Cx43 gap junctions. Note that gap junctions in some places outline B-cell borders (star is in the middle of a B cell). Immunofluorescence reaction. Bar: 10 $\mu$m.

morphogens (Loewenstein and Rose, 1992; Warner, 1992). Several lines of evidence suggest that cell growth and proliferation are inversely related to the extent of direct intercellular communication. Growth factors, oncogene activation, and tumor-promoting phorbol-esters downregulate gap-junction expression and function in cells (Trosko et al., 1993). Retinoids have the opposite effect (Hossain et al., 1989). In addition there is the observation that communication-deficient transformed cells can normalize their growth pattern after their cell-cell communication had been restored by transfecting them with connexin cDNA (Metha et al., 1991). It seems, therefore, that two of the important functions for gap-junctional communication are (1) signal propagation across coupled functional compartments in tissues and (2) serving to inhibit cell proliferation and growth that directs the cells toward differentiation.

### Communication Pathways in the Germinal Center

FDC meshworks, produced in response to antigen challenge with T-cell help, capture activated highly proliferating B cells and form round compartments called germinal centers in lymphoid tissues (MacLennan, 1994). This germinal center reaction gives rise to memory and plasma B cells specific for the antigen. Our results indicate a positive correlation between Cx43 expression and the size of the FDC network on immunization, where FDC are functionally coupled through gap junctions (Krenacs et al., 1997). From the growth regulatory function of gap junctions (Loewenstein and Rose, 1992), we hypothesize that upregulation of their expression in mature FDC may be involved in controlling the size of the follicles. Reaching a plateau in gap junction expression probably prevents follicles from further enlargement through the control of FDC growth.

Germinal centers of secondary lymphoid follicles are structurally divided into a dark zone filled with highly proliferating B centroblasts (see Figure 2) and a light zone with dense FDC meshwork enveloping maturing B centrocytes, a few germinal center T cells and macrophages (MacLennan, 1994; Thorbecke et al., 1994). B cells entering the light zone are rescued from apoptosis on the basis of their binding to FDC and the presented antigen (MacLennan and Gray, 1986; Lindhout et al., 1993). The nature of the final rescue signal blocking endonucleases in B cells is still unknown (Lindhout et al., 1995). Since FDC can communicate with B lymphocytes through gap junctions and this is the region where gap-junction expression is the highest (Krenacs and Rosendaal, 1995; Krenacs et al., 1997), one can imagine that this signal may be delivered through gap junctions. If this is so, the specific binding to the antigen presented by FDC may signal B cells to form gap junctions. The fast kinetics of gap-junction formation reflected by the short half-life (1-2 hr) of connexins (Laird et al., 1991) means that gap junctions could form in B cells within 4 hr, the predicted time needed for B-cell rescue (Ernst Lindhout, personal communication).

In the light zone of germinal centers, FDC envelope both B and T lymphocytes while forming a meshwork of interconnected cells. We showed that the members of this meshwork are functionally coupled with Cx43 gap junctions (Krenacs et al., 1997), hence they may provide a cellular internet in the germinal center between the different lymphocytes. We did not find gap junctions between FDC and T cells or between FDC and macrophages. However, there is *in vitro* evidence that T cells, probably via cytokine action or the CD40-CD40L pathway modify FDC growth and phenotype (Clark et al., 1992; Kim et al., 1994), which should have consequences on FDC-B-cell interactions.

It has to be noted that antigen-specific T cells play a pivotal role in FDC and germinal center formation, B-cell activation, and the final maturation steps of B lymphocytes (Clark and Ledbetter, 1994; Thorbecke et al., 1994). T cells act through receptor-ligand pathways represented by interactions between cytokines/growth factors, adhesion molecules, and their receptors in the target cell membrane. In recent work, however, we have confirmed the existence of another direct pathway for information exchange in the lymphoid germinal center that occurs through gap junctions (Krenacs and Rosendaal, 1995; Krenacs et al., 1997). This direct cell-cell communication may explain some unclear features of B-cell maturation and show how germinal center events are integrated. There is a lot more to do in revealing exactly how this cellular internet works and what role(s) it serves in germinal center reactions.

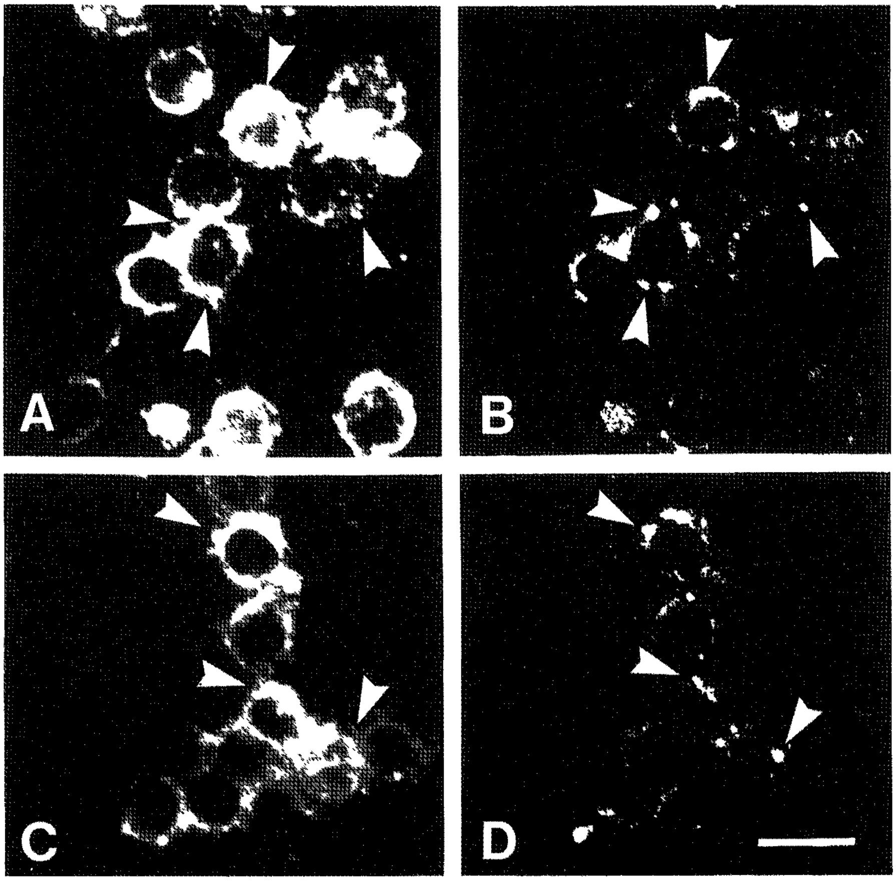

FIGURE 5 Cytospin samples of isolated tonsillar mononuclear cells simultaneously immunolabeled either for (A) IgM and (B) Cx43, or for (C) IgD and (D) Cx43. Note that some of the (A) IgM positive and (C) less IgD positive B cells express Cx43 gap-junction protein. Colocalization of pairs of antigens are indicated with arrowheads on identical cells. that is, in (A) and (B), or in (C) and (D). Bar: 10 $\mu$m.

### MATERIALS AND METHODS

### Immunohistochemistry

Human tonsils surgically removed from children with recurrent tonsillitis were used for both immunohistochemistry and culture (see later). Frozen sections 8 $\mu$m thick were fixed in 4% paraformaldehyde, then in acetone for 5 min each, and allowed to dry for 2 hr. For single-antigen detection, the sections were treated for 2 hr with rabbit anti-Cx43 (HJ; Green and Severs, 1993), desmoplakin (Dp12; gift from A.I. Magee, National Institute for Medical Research, Mill Hill, London), Ki-67, IgM, and IgD antibodies, or with mouse anti-Cx43 (Zymed, San Francisco; clone: CX-1B1), CD21, and CD35 antibodies, respectively. The specific binding sites were revealed with either biotinylated goat anti-rabbit Ig-s, or anti-mouse Ig-s, followed by the use of a FITC-conjugated Streptavidin reagent, for 1 hr each. For simultaneous antigen detection, pairs of rabbit and mouse antibodies were applied in mixtures for 2 hr followed by a mixture of TRITC-conjugated swine anti-rabbit Ig-s and FITC-conjugated goat anti-mouse Ig ($Fab'_2$) for 1 hr. All antibodies were from DAKO (Glostrup, Denmark), except where otherwise noted.

### FDC-B-Cell Cultures

The tonsils were minced and low-density FDC-rich germinal center B-cell fractions were separated as described by Lindhout et al. (1993). Briefly, tissue pieces were first digested in a mixture of Collagenase IV and DNase in Iscove's modified Dulbecco's medium. From the supernatants, cell fractions were separated on a BSA gradient (cells between the interfaces of 2.5% and 5% BSA were used) followed by Percoll gradient centrifugation. The cells of <1060 mg/ml density were cultured on 22 × 24-mm coverslips in IMDM supplemented with 0.1% gentamycin and 10% fetal calf serum. Cell cultures were fixed onto the coverslips and immunostained in the same ways as the frozen sections (see earlier) at any time points of the investigation between 2 to 24 hr. Immunostaining for immunoglobulins and Cx43 was carried out in freshly separated, low-density cell fractions too.

### *Acknowledgements*

We are grateful to Ernst Lindhout and Maike van Dartel for help in cell culture work and for their valuable comments in the interpretation of the results. We also thank The Wellcome Trust in England and the OMFB-British Council GB-26/95 and OTKA T022277 in Hungary for supporting this work.

### *References*

Alves L.A., Campos de Carvalho A.C., Cirne Lima E.O., Rocha e Souza C.M., Dardenne M., Spray D.C., and Savino W. (1995). Functional gap junctions in thymic epithelial cells are formed by connexin 43. Eur. J. Immunol. **25**: 431-437.

Bruzzone R., White T.W., and Goodenough D.A. (1996). The cellular internet: On-line with connexins. BioEssays **18**: 709-718.

Clark E.A., Grabstein K.H., and Shu G.L. (1992). Cultured human follicular dendritic cells. Growth characteristics and interactions with B lymphocytes. J. Immunol. **148**: 3327-3335.

Clark E.A. and Ledbetter J.A. (1994). How B and T cells talk to each other. Nature **367**: 425-428.

Green C.R., and Severs N.J. (1993). Distribution and role of gap junctions in normal myocardium and human ischaemic heart disease. Histochemistry **99**: 105-120.

Granot I., and Dekel N. (1994). Phosphorylation and expression of connexin-43 ovarian gap junction protein are regulated by luteinizing hormone. J. Biol. Chem. **269**: 30502-30509.

Hossain M.Z., Wilkens L.R., Metha P.P., Loewenstein W.R., and Bertram J.S. (1989). Enhancement of gap junctional communication by retinoids correlates with their ability to inhibit neoplastic transformation. Carcinogenesis **10**: 1743-1748.

Kim H-S., Zhang X., and Choi Y.S. (1994). Activation and proliferation of follicular dendritic cell-like cells by activated T lymphocytes. J. Immunol. **153**: 2951-2961.

Krenacs T., and Rosendaal M. (1995). Immunohistological detection of gap junctions in human lymphoid tissue: Connexin43 in follicular dendritic and lymphoendothelial cells. J. Histochem. Cytochem. **43**: 1125-1137.

Krenacs T., Rosendaal M., van Dartel M., and Lindhout E. (1997). Direct cell-cell communication in lymphoid follicles: Connexin43 gap junctions functionally couple follicular dendritic cells and follicular dendritic cells with B lymphocytes. Eur. J. Immunol. **27**: 1489-1497.

Kumar N.M., and Gilula N.B. (1996). The gap junction communication channel. Cell **84**: 381-388.

Laird D.W., Puranam K.L., and Revel J.P. (1991). Turnover and phosphorylation dynamics of connexin43 gap junction protein in cultured cardiac myocytes. Biochem. J. **273**: 67-72.

Lindhout E., Lakeman A., and de Groot C. (1995). Follicular dendritic cells inhibit apoptosis in human B lymphocytes by a rapid and irreversible blockade of preexisting endonuclease. J. Exp. Med. **181**: 1985-1995.

Lindhout E., Mevissen M.L.C.M., Kwekkeboom J., Tager J.M., and de Groot C. (1993). Direct evidence that human follicular dendritic cells rescue germinal center B cells from death by apoptosis. Clin. Exp. Immunol. **91**: 330-336.

Loewenstein W.R., and Rose B. (1992). The cell-cell channel in the control of growth. Semin. Cell Biol. **3**: 59-79.

MacLennan I.C.M. (1994). Germinal centrers. Ann. Rev. Immunol. **12**: 117-139.

MacLennan I.C.M., and Gray D. (1986). Antigen-driven selection of virgin and memory B cells. Immunol. Rev. **91**: 61-85.

Makowski L., Casper D.L.D., Phillips W.C., and Goodenough D.A. (1977). Gap junction structures. J. Cell Biol. **74**: 629-645.

Metha P.P., Hotz-Wagenblatt A., Rose B., Shalloway D., and Loewenstein W.R. (1991). Incorporation of the gene for the cell-cell channel protein into transformed cells leads to normalization of growth. J. Membr. Biol. **124**: 207-225.

Miller S.M., Garfield R.E., and Daniel E.E. (1989). Improved propagation in myometrium associated with gap junctions during parturition. Am. J. Physiol. **256**: C130-C141.

Nathanson M.H., Burgstahler A.D., Mennone A., Fallon M.B., Gonzales C.B., and Saez J.C. (1995). $Ca^{2+}$ waves are organized among hepatocytes in the intact organ. Am. J. Physiol. **269**: C167-C171.

Rook M.B., de Jonge B., Jongsma H.J., and Masson Pevet M.A. (1990). Gap junction formation and functional interaction between neonatal rat cardiocytes in culture: A correlative physiological and ultrastructural study. J. Membr. Biol. **118**: 179-192.

Rosendaal M., Gregan A., and Green C.R. (1991). Direct cell-cell communication in the blood-forming system. Tissue Cell **23**: 457-470.

Rosendaal M., Green C.R., Rahman A., and Morgan D. (1994). Upregulation of connexin 43 gap junction network in haemopoietic tissue before the growth of stem cells. J. Cell Sci. **107**: 29-37.

Saez J.C., Conner J.A., Spray D.C., and Bennett M.V.L. (1989). Hepatocyte gap junctions are permeable to the second messenger, inositol 1,4,5-triphosphate, and to calcium ions. Proc. Natl. Acad. Sci. USA **86**: 2708-2712.

Thorbecke G.J., Amin A.R., and Tsiagbe V.K. (1994). Biology of germinal centres in lymphoid tissue. FASEB J. **8**: 832-840.

Trosko J.E., Madhukar B.V., and Chang C.C. (1993). Endogenous and exogenous modulation of gap junctional intercellular communication: Toxicological and pharmacological implications. Life Sci. **53**: 1-19.

Vozzi C., Ullrich S., Charollais A., Philippe J., Orci L., and Meda P. (1995). Adequate connexin mediated coupling is required for proper insulin secretion. J. Cell. Biol. **131**: 1561-1572.

Warner A. (1992). Gap junctions in development—A perspective. Semin. Cell Biol. **3**: 81-91.

